# Frontal cortex electrophysiology in reward- and punishment-related feedback processing during advice-guided decision making: An interleaved EEG-DC stimulation study

**DOI:** 10.3758/s13415-018-0566-8

**Published:** 2018-01-29

**Authors:** Miles Wischnewski, Harold Bekkering, Dennis J. L. G. Schutter

**Affiliations:** Donders Institute for Brain, Cognition and Behaviour, Radboud University Nijmegen, Montessorilaan 3, Room B.01.21, 6525 HR Nijmegen, The Netherlands

**Keywords:** Advice information, Decision making, Electroencephalogram, Feedback processing, Transcranial direct current stimulation

## Abstract

During decision making, individuals are prone to rely on external cues such as expert advice when the outcome is not known. However, the electrophysiological correlates associated with outcome uncertainty and the use of expert advice are not completely understood. The feedback-related negativity (FRN), P3a, and P3b are event-related brain potentials (ERPs) linked to dissociable stages of feedback and attentional processing during decision making. Even though these ERPs are influenced by both reward- and punishment-related feedback, it remains unclear how extrinsic information during uncertainty modulates these brain potentials. In this study, the effects of advice cues on decision making were investigated in two separate experiments. In the first experiment, electroencephalography (EEG) was recorded in healthy volunteers during a decision-making task in which the participants received reward or punishment feedback preceded by novice, amateur, or expert advice. The results showed that the P3a component was significantly influenced by the subjective predictive value of an advice cue, whereas the FRN and P3b were unaffected by the advice cues. In the second, sham-controlled experiment, cathodal transcranial direct current stimulation (ctDCS) was administered in conjunction with EEG in order to explore the direct contributions of the frontal cortex to these brain potentials. Results showed no significant change in either advice-following behavior or decision times. However, ctDCS did decrease FRN amplitudes as compared to sham, with no effect on the P3a or P3b. Together, these findings suggest that advice information may act primarily on attention allocation during feedback processing, whereas the electrophysiological correlates of the detection and updating of internal prediction models are not affected.

**Electronic supplementary material:**

The online version of this article (10.3758/s13415-018-0566-8) contains supplementary material, which is available to authorized users.

Decision making is a multifaceted process associated with evaluating and selecting among a finite set of alternatives on the basis of probability and outcome (Lee, 2013). Both implicit and explicit forms of knowledge are used to reduce uncertainty and maximize the likelihood of making the correct choice. An important source of explicit knowledge that guides decision making during uncertainty comes from expert advice. This is advice that is subjectively perceived as a reliable predictor of the desired outcome (Sniezek, Schrah, & Dalal, 2004), which has been further illustrated by results showing that even good decision-makers remain biased toward the opinions of experts (Cook, den Ouden, Heyes, & Cools, 2014; Harvey & Fischer, 1997). The usefulness of advice is determined by its predictive value in terms of rewards and punishments (Bonaccio & Dalal, 2006). Yet how advice affects the cortical processing underlying decision making is still poorly understood.

It is assumed that during feedback processing, an internal prediction model is used to evaluate the current feedback. On the basis of errors in the prediction, this model can be further refined. Electroencephalography (EEG) studies have identified event-related potential (ERP) components that are associated with different stages of feedback processing (Baker & Holroyd, 2011; Cavanagh, Masters, Bath, & Frank, 2014; Enriquez-Geppert, Konrad, Pantev, & Huster, 2010). First, an internal prediction model detects a mismatch between the expected and actual outcomes. The fronto-central midline feedback-related negativity (FRN) is associated with such error detection (Holroyd & Coles, 2002; Ullsperger, Fischer, Nigbur, & Endrass, 2014). Furthermore, several studies have indicated that the FRN is affected by the valence and magnitude of rewards, as well as by the context in which the rewards are presented (Bellebaum, Polezzi, & Daum, 2010; Holroyd, Larsen, & Cohen, 2004; Wu & Zhou, 2009).

Source localization studies have indicated that the neural generator of the FRN lies in the anterior cingulate cortex (ACC; Bocquillon et al., 2014; Hauser et al., 2014; Ullsperger et al., 2014). In addition, functional magnetic resonance imaging (fMRI) studies have supported the importance of the ACC in reward and punishment processing, together with the orbitofrontal cortex (OFC) and ventral striatum (Beckmann, Johansen-Berg, & Rushworth, 2009; Rogers et al., 2004). There is increasing evidence that the neural activity in these regions during reward processing is modulated by the presence of expert information (Engelmann, Capra, Noussair, & Berns, 2009; Engelmann, Moore, Capra, & Berns, 2012; Meshi, Biele, Korn, & Heekeren, 2012; Tomlin, Nedic, Prentice, Holmes, & Cohen, 2013). Hemodynamic activity in the OFC has been shown to increase when advice is more likely to change one’s initial opinion in favor of following the expert’s opinion (Meshi et al., 2012). Furthermore, the OFC has been shown to reflect the subjective value of rewards and external information (Padoa-Schioppa & Cai, 2011; Peters & Büchel, 2010). These results indicate that the OFC is involved in processing the subjective valuation of advice cues, in which seemingly more informative cues are associated with increased OFC activity and, hence, increased following behavior (Meshi et al., 2012). In contrast, Suen, Brown, Morck, and Silverstone (2014) showed increased ACC activity when financially disadvantageous expert advice was opposed, providing a possible way to override following advice. Therefore, whether advice is followed may depend on a balance between the urge to follow experts, mediated by OFC activity, and the actual benefits of following advice, mediated by ACC activity. In addition to cortical structures, this balance determining the subjective value of a cue has also been associated with activity in the ventral striatum (Meshi et al., 2012).

Following the detection of a mismatch between the expected and actual outcomes the prediction model is updated to make the model more accurate for future feedback. A parietal positive deflection that can be observed between 300 and 600 ms (P300) after reward- and punishment-related feedback is associated with these processes (Goldstein et al., 2006). The P300 component is associated with attention allocation and consists of two subcomponents, the P3a and P3b (Polich, 2007). It has been proposed that the P3a reflects a process of novelty detection (Polich, 2007). Studies have indicated that the amplitude of the P3a is influenced by the expectedness of feedback (Donchin & Coles, 1988; Donchin, Ritter & McCallum, 1978), as well as by the magnitude of the reward or punishment (Sato et al., 2005; Wu & Zhou, 2009). On the basis of these observations, the allocation of attention toward relevant information accompanies a higher P3a amplitude (Polich, 2007). In accordance, neuroimaging studies have related the P300 to the fronto-parietal attention network (Bengson, Kelley, & Mangun, 2015; Pfabigan et al., 2014). Fronto-parietal network activity, it is proposed, is directly influenced by the amount of uncertainty during decision making (Kopp et al., 2016; Paulus et al., 2001). Expert advice can increase confidence in a decision, especially during uncertain situations (Bonaccio & Dalal, 2006). However, it is unknown whether the predictive value of advice modulates attention allocation, and consequently P3a amplitudes, during reward and punishment feedback. The P3b component has been suggested to underlie the adaptation of behavior in subsequent trials by means of updating one’s internal prediction model on the basis of current reward or punishment feedback (Fischer & Ullsperger, 2013; Polich, 2007). Studies have indicated that changes in behavior associated with learning and optimizing decision making are paralleled by larger P3b amplitudes (Chase, Swainson, Durham, Benham, & Cools, 2011; Fischer & Ullsperger, 2013).

The FRN, P3a, and P3b are thus thought to reflect different aspectsunderlying feedback processing. FMRI studies have provided evidence that activity in the networks associated with these ERPs is influenced by advice during decision making (Meshi et al., 2012). However, the extent to which advice actually affects these electrophysiological components of feedback processing remains unclear. Although some studies have investigated the ERP components directly related to the presentation of an advice cue (Chen, Wu, Tong, Guan, & Zhou, 2012; Kim, Liss, Rao, Singer, & Compton, 2012; Kimura & Katayama, 2013; Shestakova et al., 2013; Trautmann-Lengsfeld & Herrmann, 2013; Yu & Sun, 2013), to date no study has investigated the effects of advice cues on the ERP components of subsequent feedback processing. If people are to accurately use advice during decision making, the advice needs to be validated and its usefulness—that is, whether the advice is predictive of subsequent gains or losses—needs to be determined. It is therefore possible that feedback- and attention-related electro-cortical components are susceptible to advice cues.

The present study consisted of two experiments aimed at delineating the relation between advice cues and the processes related to feedback and attention. In the first experiment, the effects of advice cues on performance and ERPs were investigated during a forced choice reward–punishment task. We hypothesized that expert cues would be perceived as more predictive than nonexpert cues. Furthermore, we expected that this difference in perceived predictiveness would be reflected in the subsequent feedback-processing ERP components. Specifically, feedback following more predictive cues would be of more importance for making successful choices. Therefore, we hypothesized that advice cues viewed as being more predictive would increase mismatch detection and direct attention toward the feedback, as revealed by larger FRN and P300 amplitudes.

In the second experiment, cathodal transcranial direct current stimulation (ctDCS) targeting the OFC was applied during the forced choice reward–punishment task, in order to directly manipulate decision making on the basis of advice cues and the associated feedback-related brain potentials. Transcranial direct current stimulation (tDCS) has been shown to be effective in modulating both cortical physiological activity (Nitsche et al., 2003; Nitsche & Paulus, 2000, 2001) and cognitive performance (for a review, see Kuo & Nitsche, 2015). Because the OFC is related to processing of the subjective valuation of advice cues (Meshi et al., 2012), we hypothesized that ctDCS-related interference with OFC activity would decrease the subjective biases toward the advice cues. As a result, the percentage following of advice cues in this case would be closer to chance level. Additionally, we explored the effects of online ctDCS on feedback-related ERPs. Because our hypothesis in the first experiment had stated that increased FRN and P300 amplitudes are related to increased advice following, we expected to find a decrease in FRN and P300 amplitudes during ctDCS.

## Experiment 1

### Materials and method

#### Participants

Twenty-one right-handed participants with (corrected-to-)normal vision and no history of neurological or psychiatric disorders participated in Experiment 1 (12 female, nine males; mean age ± *SD*: 22.67 ± 3.18 years). The study protocol was approved by the Committee on Research Involving Human Subjects of the Radboud University Medical Centre.

#### Experimental design and procedure

**Fig. 1:**
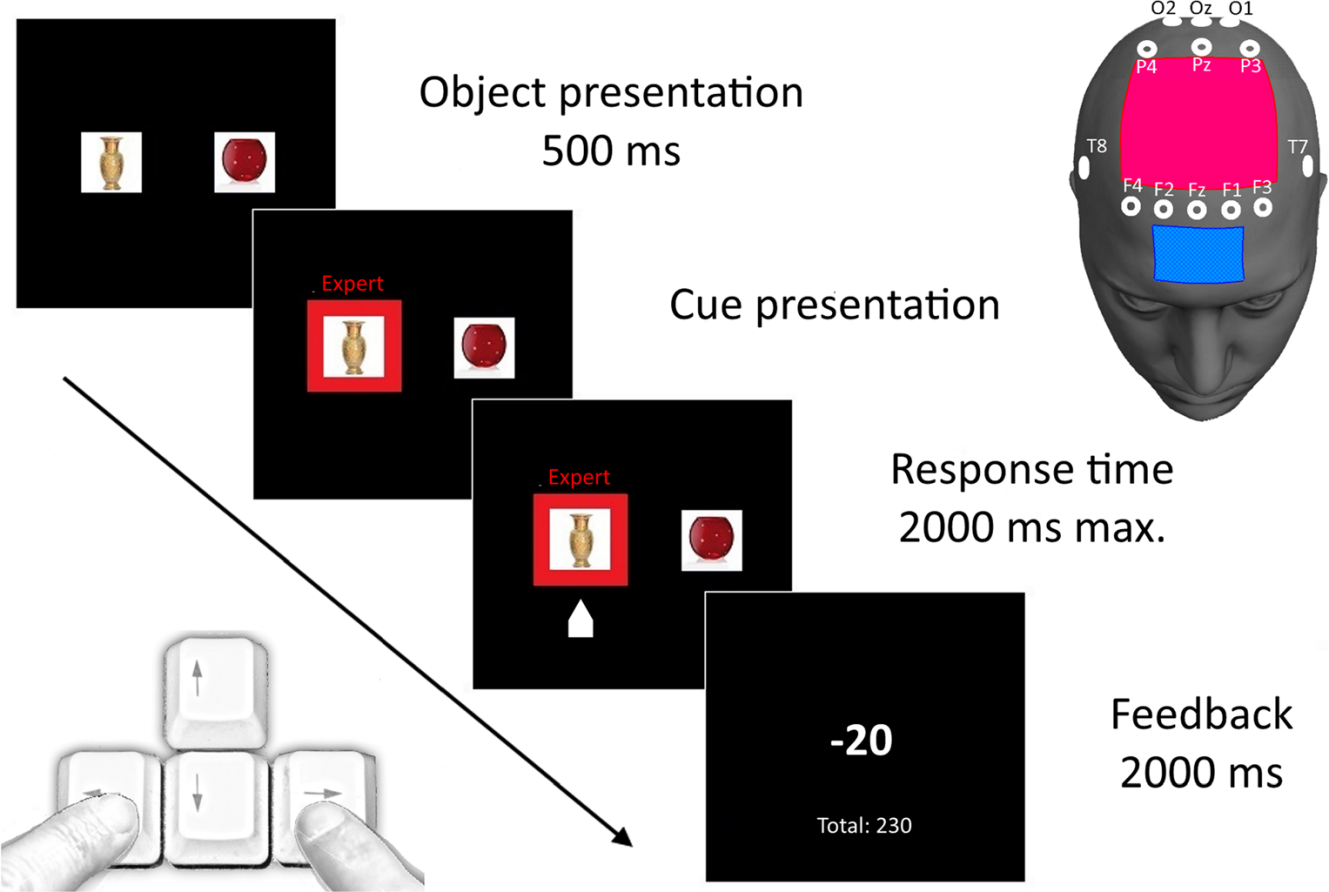
(Left) Overview of one trials. In total, 120 trials were presented, with a jittered intertrial interval of 100–1,000 ms. The total duration of the task was approximately 10 min. (Right) EEG setup of Experiment 1 (electrodes), and tDCS-EEG setup of Experiment 2 (blue: cathode, red: anode)

In each trial of the decision-making task, two neutral objects of the same type (vases) were presented on a black screen (22-in., 30 × 48 cm; resolution: 1,024 × 768), and participants were asked to indicate which was more expensive. The participants were placed approximately 80 cm from the screen, and the objects (resolution 350 × 250 pixels) were presented 5 cm to the left and right of the screen’s center point, on a white background. In every trial different vases were shown (a total of 240 vases), so as to prevent learning effects. After the objects had appeared, one of three advice cues was randomly presented, indicated by a red frame (1-cm width) surrounding the picture. Participants were informed that the advice cue represented the choice of a group of participants from a previous study. Three types of cues were used—“novices,” “amateurs,” and “experts”—with the labels being shown above the red frame. The level of expertise was manipulated by informing participants that the experts had attained high scores on this task, whereas the novices had low scores. However, participants were free to use any decision strategy. Although advice information purposely implied that following the expert cues was better than following the novice cues, in reality the predictive value of each cue was at chance level. The objects were shown for maximally 2,500 ms, and the cue appeared after the first 500 ms. This means that the participants had a maximum of 2,000 ms to make a decision by pressing either the left or the right button with their index finger (Fig. 1). Subsequently, the participants received points that reflected monetary rewards. The points ranged from – 40 (punishment) to + 50 (reward) in steps of 10. When the participants did not respond within 2,000 ms, a message with the text “faster” appeared. The outline of a single trial is shown in Fig. 1. To prevent participants from realizing that the advice cue information was random, they were informed that the points that they received were relative to the points they would have received if they had opted for the alternative. Since the number of points for this alternative was not presented, participants were kept uncertain about the actual correctness of their choice. A total of 120 trials were presented, with 40 trials for each advice cue and an intertrial interval of 100–1,000 ms. Data from the behavioral task were stored for offline analysis using the Presentation software (Neurobehavioral systems, Berkeley, CA, USA).

To study the underlying ERP components of advice information processing, EEG measurements were performed during the task. After participants had been informed about the task, EEG electrodes were placed. Then participants performed the decision-making task for approximately 10 min, after which the electrodes were removed and participants were debriefed.

#### Electroencephalography recording

EEG was recorded continuously during the task using an online 0.1- to 70-Hz band-pass filter with a sampling rate of 1000 Hz on a passive 64-channel EASYCAP with a transcranial magnetic stimulation multitrode system (EASYCAP GmbH, Herrsching, Germany). Recordings were made from a selection of 13 resin-covered sintered Ag/AgCl electrodes (F3, F1, Fz, F2, F4, P3, Pz, P4, T7, T8, O1, Oz, O2), shown in Fig. 1. The reference electrode was positioned on the left mastoid, and the ground electrode was placed at POz. Furthermore, a vertical electro-oculogram was recorded from electrodes above and below the left eye, and a horizontal electro-oculogram was recorded from electrodes at the outer canthi of both eyes. Raw EEG data were recorded and stored for offline analysis using BrainVision Analyzer 2.0 (Brain Products GmbH, München, Germany).

#### Data reduction and processing

##### Advice information processing

For each participant, the percentages of following the novice, amateur, and expert cues were calculated. To determine how informative each cue was experienced to be by the participants, the subjective predictive value (SPV) was calculated. This value is the absolute difference between the percentage following and chance level (50%). Therefore, the SPV is a value between 0 and 50, with larger numbers representing higher subjective information value. For example, if a cue is followed on either 100% or 0% of trials, the SPV would be 50. This would mean that these cues were highly informative, since participants either followed or opposed the cue at all times, whereas an SPV of 0 would imply that the cue was uninformative to the participant, and thus was followed at chance level.

##### ERP data analysis

All EEG recordings were offline band-pass filtered between 1.5 and 30 Hz (48 dB/Octave) and referenced to the mastoid. ERPs of 1,000 ms were segmented time-locked to the moment at which participant had received feedback immediately following the decision. Epochs were baseline-corrected using the – 100-ms to 0-ms window. Ocular artifacts were controlled using the Gratton and Coles algorithm (Gratton, Coles, & Donchin, 1983). Additional artifacts were removed automatically if the peak-to-peak differences exceeded 100 *μ*V or were below 0.5 *μ*V, followed by visual inspection of the data. The remaining epochs were averaged separately for (1) reward versus punishment and (2) novice versus amateur versus expert trials. All ERP results were analyzed at the Pz electrode. This electrode was chosen in order to compare the findings with experiments in which only the Pz electrode was investigated (see the ERP Data Analysis section of Exp. 2 and Supplementary Table 1). The ERPs recorded at the other channels are displayed in Supplementary Figs. S1 and S2.

#### Statistical analysis

Expert information processing was measured by the number of advice cues followed during the decision-making task. Generalized linear model (GLM) repeated measures analyses of variance (ANOVAs), with the dependent variables percentage following cues and reaction time, were used to investigate how the advice cues influenced decision making. Significant results were followed by Bonferroni-corrected paired-samples *t* tests. Additionally, in a post hoc test, the SPV values were tested against a test value of 0 (indicating no predictive value).

ERP components were investigated using GLM repeated measures ANOVAs in the time windows of the FRN (200–300 ms; Gentsch, Ullsperger, & Ullsperger, 2009), the P3a (350–400 ms; Muller-Gass, Macdonald, Schrönger, Sculthorpe, & Campbell, 2007), and the P3b (450–600 ms; Calvo & Beltrán, 2014). Significant results were followed by an exploratory analysis in which the time course of a significant difference was made by plotting the *p* value every 4 ms within the investigated time window.

All statistical analyses were performed using IBM SPSS 22.0, and the statistical level of significance was set to *α* < .05 (two-tailed). All analyses were checked for normality, and Mauchly’s test was used to examine the assumption of sphericity. A Greenhouse–Geisser correction was used if this assumption was violated. All averages are represented as means ± *SEM*s.

### Results

#### Advice cue processing

**Fig. 2:**
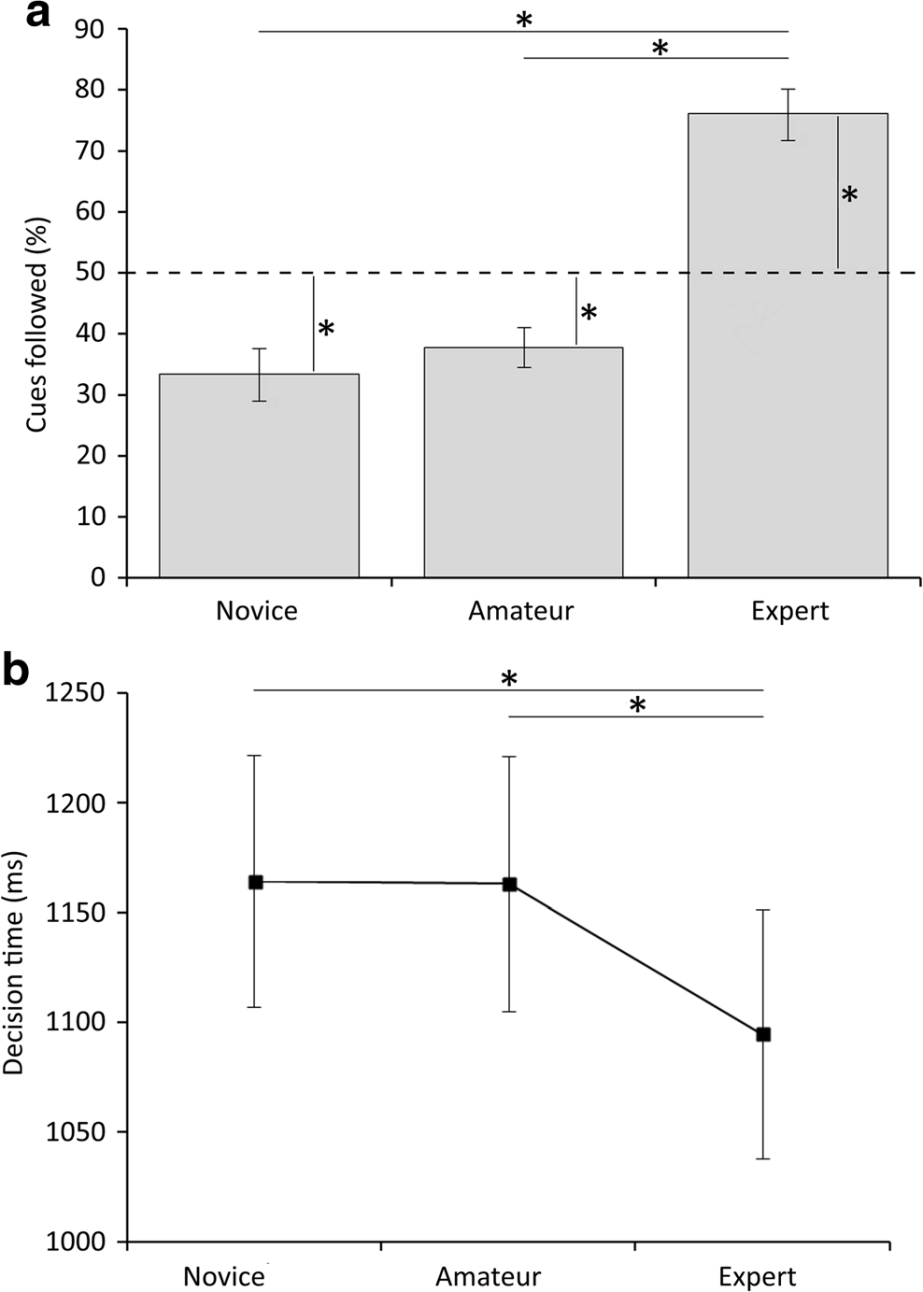
(**a**) Percentages of advice following for novice, amateur, and expert cues in Experiment 1. Percentages following were compared between different cue types and to chance level (50%). (**b**) Decision times for each of the three advice cues. Asterisks indicate significant results after Bonferroni correction (*p* < .05)

Participants chose the left vase in 50.59 ± 1.16% of trials, and the right vase in 48.89 ± 1.09% of trials, which did not differ significantly [*t*(20) = 0.76, *p* = *.*45], and responses occurred too late in 0.51 ± 0.30% of trials. A significant difference was observed between percentages following the three advice cues [*F*(2, 40) = 31.91, *p* < .001]. On average, participants followed novices in 33.41 ± 4.23% of trials, amateurs in 37.80 ± 3.26%, and experts in 76.10 ± 4.22%. Bonferroni-corrected pairwise comparisons revealed that experts were followed significantly more than both novices [*t*(20) = 6.11, *p* < .001] and amateurs [*t*(20) = 6.31, *p* < .001], but the percentages following novices and amateurs were not different (Fig. 2a). Furthermore, we tested whether the SPVs—that is, the absolute differences from chance level (50%)—were significantly greater than zero. Consequently, Bonferroni-corrected one-sample *t* tests were performed, and the SPVs of all cues [novice: SPV = 16.59, *t*(20) = 3.82, *p* = .003; amateur: SPV = 12.20, *t*(20) = 3.75, *p* = .003; expert: SPV = 26.10, *t*(20) = 6.18, *p* < .001] were significantly different from chance level (Fig. 2a). Expert cues were subjectively perceived as being most predictive, whereas amateur cues were seen as least predictive. Interestingly, the SPVs were significantly positively correlated between all three conditions (novice–amateur, *r* = .507, *p* = .019; novice–expert, *r* = .529, *p* = .014; amateur–expert, *r* = .508, *p* = .019). This suggest that participants who relied more on advice information in one condition also relied more on advice information in another condition.

Decision times differed significantly between the advice cue categories, as well [*F*(2, 20) = 19.00, *p* < .001]. Bonferroni-corrected pairwise comparisons revealed that during expert trials decisions were made significantly faster than during either novice trials [*t*(20) = 7.02, *p* < .001] or amateur trials [*t*(20) = 4.60, *p* = .001]. No difference was observed between novice and amateur trials [*t*(20) = 0.08, *p* = .936] (Fig. 2b). Furthermore, no correlation between the averaged decision times and averaged SPVs was found (*r* = – .186, *p* = .420).

#### Reward and punishment ERPs

**Fig. 3:**
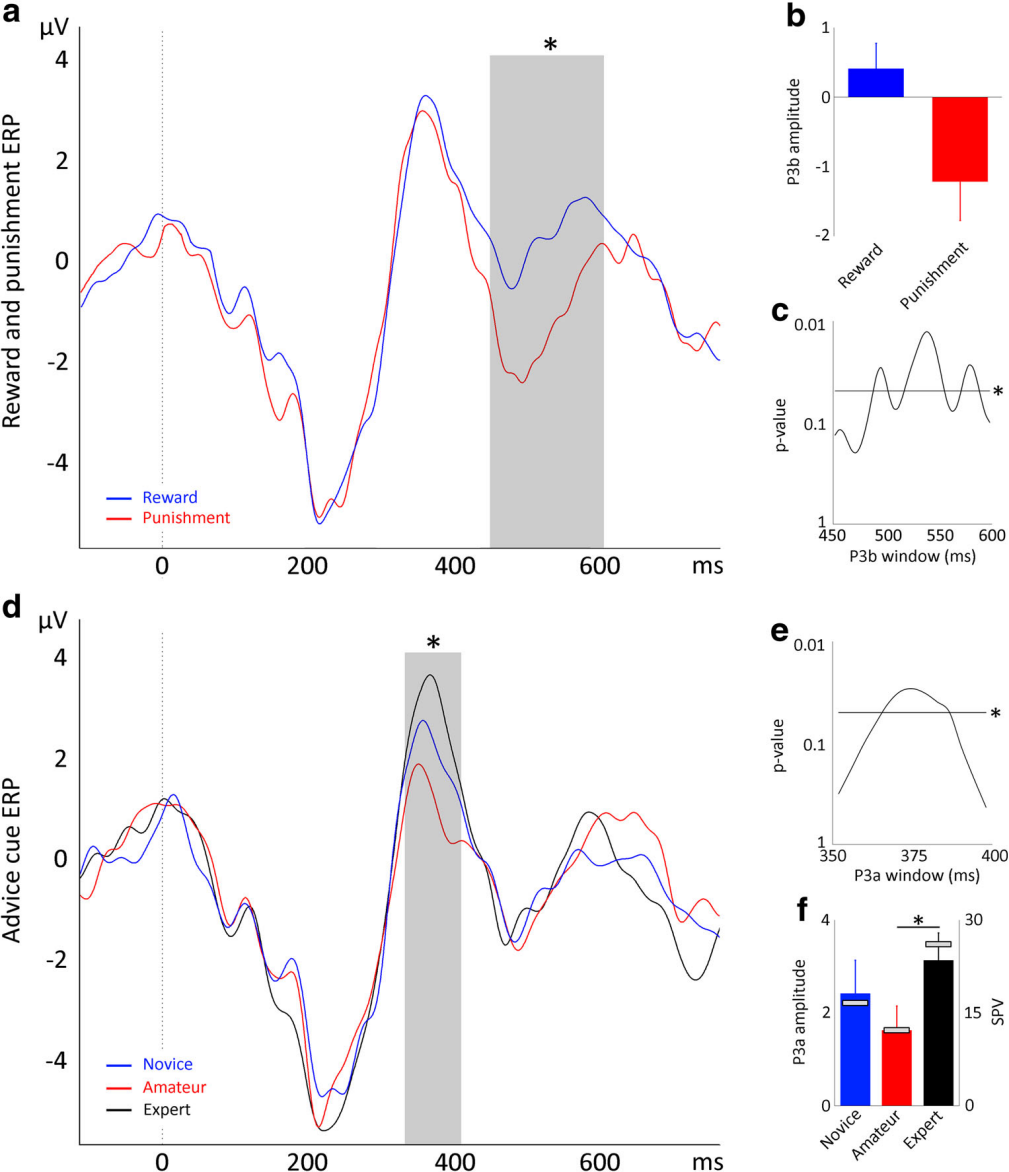
(**a**) ERP responses time-locked to reinforcement feedback immediately after decision making for reward and punishment trials in Experiment 1. (**b** & **c**) P3b amplitudes (**b**) and time course (**c**) in the interval 450–600 ms after feedback onset for reward and punishment trials. (**d**) ERP responses time-locked to reinforcement feedback immediately after decision making for different advice cues in Experiment 1. (**e**) Time course of the differences between advice cues within the P3a. (**f**) P3a amplitudes 350–400 ms after feedback onset for advice cues (left *y*-axis). The gray bars in panel F reflect the SPV for each cue (right *y*-axis). Asterisks indicate significant effects (*p* < .05)

A significant difference for the P3b component was observed between reward and punishment trials [Pz, *F*(1, 20) = 5.99, *p* = .024; Figs. 3a, b], specifically in a time window of 492–588 ms (Fig. 3c). No significant difference between reward and punishment trials was observed for the FRN [Pz, *F*(1, 20) = 0.01, *p* = .965] or the P3a [Pz, *F*(1, 20) = 0.01, *p* = .934]. Similar results were observed for electrodes P3 and P4, but no differences between reward and punishment trials were found in other electrodes (Supplementary Fig. S1).

#### Advice cue ERPs

Analysis of ERPs segmented for different advice cues revealed a trend toward significance at the P3a component [*F*(2, 40) = 3.30, *p* = *.*047; Fig. 3d]. Indeed, analysis of the time points within the P3a window revealed a significant effect between 366 and 386 ms (Fig. 3e). Bonferroni-correct post hoc comparisons revealed a significantly larger P3a amplitudes in response to expert than to amateur cues [*t*(20) = 2.73, *p* = *.*039; Fig. 3f]. The novice–expert and novice–amateur differences in ERP signals did not reach significance. The modulation of the P3a component concurred with the differences in SPVs (Fig. 3f). No effect of advice cues was found on the FRN [*F*(2, 40) = 2.43, *p* = *.*101] or the P3b [*F*(2, 40) = 0.20, *p* = *.*822].

## Experiment 2

Indeed, Reinhart and Woodman (2014) have shown that FRN amplitudes are decreased and error positivities are increased after offline cathodal tDCS (ctDCS), and that both are associated with reduced behavioral adjustments after errors. Here we explored the effects of online ctDCS on feedback-related ERPs. On the basis of Experiment 1, we therefore expected P3a amplitudes to be decreased. Furthermore, in accordance with the findings of Reinhart and Woodman, a decrease in FRN amplitudes was also expected.

### Materials and method

#### Participants

Thirty right-handed participants took part in Experiment 2 (19 female, 11 male; mean age ± *SD*: 22.13 ± 3.50 years), none of whom had participated in Experiment 1. All participants had normal or corrected-to-normal vision and no history of neurological or psychiatric disorders.

#### Experimental design and procedure

During this experiment the same behavioral task was utilized, during which sham and cathodal stimulation were applied in a double-blind within-subjects design. Because participants performed in two sessions, the original behavioral task (with vases) was presented once, and in the other session the same behavioral task was used with other neutral objects (lamps). The order of the stimulation and behavioral task objects was randomized. To control for effects of diurnal rhythms, both sessions started at the same time of day (Ridding & Ziemann, 2010). To prevent carryover effects, the sessions were spaced 7 days apart (Nitsche & Paulus, 2001).

The procedure was similar to that of Experiment 1, with the exception that ctDCS was applied during the task, which was turned on 4 min prior to the beginning of the task and lasted throughout the task’s duration.

#### Transcranial direct current stimulation

TDCS was delivered with a battery-driven constant-current stimulator (DC stimulator, NeuroConn GmbH, Ilmenau, Germany), using two rubber electrodes in saline-soaked sponges. Additional conductive gel was used to improve conductivity between the electrodes and the scalp and to avoid bridging between EEG sensors. In addition, the EEG sensors were protected by a resin cover. The active tDCS electrode (5 × 7 cm) was placed horizontally over Fpz, whereas the reference electrode (10 × 10 cm) was placed over Cz (Manuel et al., 2014) Stimulation was applied at an intensity of 1.0 mA (current density active: 0.029 mA/cm^2^; reference: 0.01 mA/cm^2^), which is within the bounds of tDCS safety guidelines (Nitsche et al., 2008). The electrode impedance was kept below 10 kΩ during the stimulation period.

Active ctDCS was applied for approximately 14 min from 4 min before the onset of the behavioral task, to ensure that the effects of stimulation would be maximal at the start of the behavioral task (Manuel, David, Bikson, & Schnider, 2014; Nitsche & Paulus, 2000). During the fade-in period, the current was gradually increased over 30 s (Zaehle, Sandmann, Thorne, Jäncke, & Herrmann, 2011). During sham tDCS, a current was applied for only 30 s using the same montage as in the active ctDCS condition, which has been shown to successfully blind participants (Gandiga, Hummel, & Cohen, 2006). All participants underwent both the active and sham stimulation conditions. After each session, participants were asked to indicate whether the tDCS had felt unpleasant. Also, to ensure successful blinding, participants were asked to indicate in which of the two conditions they had received active stimulation.

#### Electroencephalography recording

The EEG apparatus, setup, and recording were the same as in Experiment 1.

#### ERP data analysis

EEG data preparation and analysis were performed in the same way as in Experiment 1. The preprocessing steps were standardized across the two experiments. In both experiments the same criteria for removing artifacts were used (automatic removal of peak-to-peak differences above 100 *μ*V and below 0.5 *μ*V, followed by visual inspection). This was done in order to avoid any quality differences in the recorded EEG signals between the experiments. Due to the proximity between the EEG and tDCS electrodes, a considerable amount of clipping or high-intensity noise was observed in frontal electrodes. The participant’s data from a single channel were excluded if more than 50% of the epochs were rejected. As a result, the Fz electrode was excluded from further analyses because 23 participants (77%) met this criterion. For electrode Pz, the data from seven participants were excluded, and the data of the remaining 23 participants were analyzed and are reported here. To compare data quality between the sham and active ctDCS conditions, the percentages of removed segments, based on the above-mentioned artifact removal criteria, were compared. From the remaining 23 participants, on average 3.83 ± 1.98% of the segments were removed in the sham condition, and 4.56 ± 1.56% of segments were removed in the ctDCS condition. A within-subjects ANOVA did not reveal a significant difference in the percentages of trials removed [*F*(1, 22) = 0.10, *p* = .755]. For the main analysis, the artifact-free epochs were averaged separately for (1) reward versus punishment, (2) novice versus amateur versus expert, and (3) sham versus cathodal tDCS trials.

To investigate changes in oscillatory activity, an exploratory time–frequency analysis was performed by using the fast-Fourier-transformed power spectra of single-trial EEG data using complex Morlet wavelets, using 30 logarithmically increasing steps between 1.5 and 10 Hz. All ERP results were analyzed at the Pz electrode. Other electrodes of interest were not taken into account because of the large amount of data loss (Supplementary Table 1).

#### Statistical analysis

The effects of ctDCS on the percentages following cues and the reaction times were investigated using GLM repeated measures ANOVAs. To check for a test–retest effect between sessions, a repeated measures ANOVA was performed, with session number as an independent variable and the percentage following of cues as the dependent variable. All comparisons between ERP components (reward vs. punishment, “RewPun”; novice vs. amateur vs. expert, “CueType”; sham vs. real, “Stim”), as well as any interaction effects, were analyzed using GLM repeated measures ANOVA analyses for the FRN (200–300 ms), P3a (350–400 ms), and P3b (450–600 ms). For the spectral analysis of evoked power, a time window of interest between 200 and 500 ms was selected, at which the FRN and P300 were observed (Cohen, Elger, & Ranganath, 2007; Wischnewski & Schutter, 2017). Paired-samples *t* tests were performed to test the difference between sham and active ctDCS for reward and punishment trials separately in the windows of 2.5–4.0, 4.0–5.5, and 5.5– 7.0 Hz. Because this was an exploratory analysis, no multiple-comparison correction was performed.

All statistical analyses were performed using IBM SPSS 22.0, and the statistical level of significance was set to *α* < .05 (two tailed). All analyses were checked for normality, and Mauchly’s test was used to examine the assumption of sphericity. A Greenhouse–Geisser correction was used if this assumption was violated. All averages are represented as means ± *SEM*s.

### Results

The stimulation was tolerated well, and only one participant reported mild discomfort (a constant itching sensation). Due to tDCS-related artifacts, the ERP data from electrode Pz could not be analyzed in seven participants (Supplementary Table 1). Other electrodes of interest were not taken into account because of large amounts of data loss. The blinding of real versus sham tDCS was successful (43.3% correct; *χ*^2^ = 1.80, *p* = *.*180).

#### Effect of ctDCS on advice processing

**Fig. 4:**
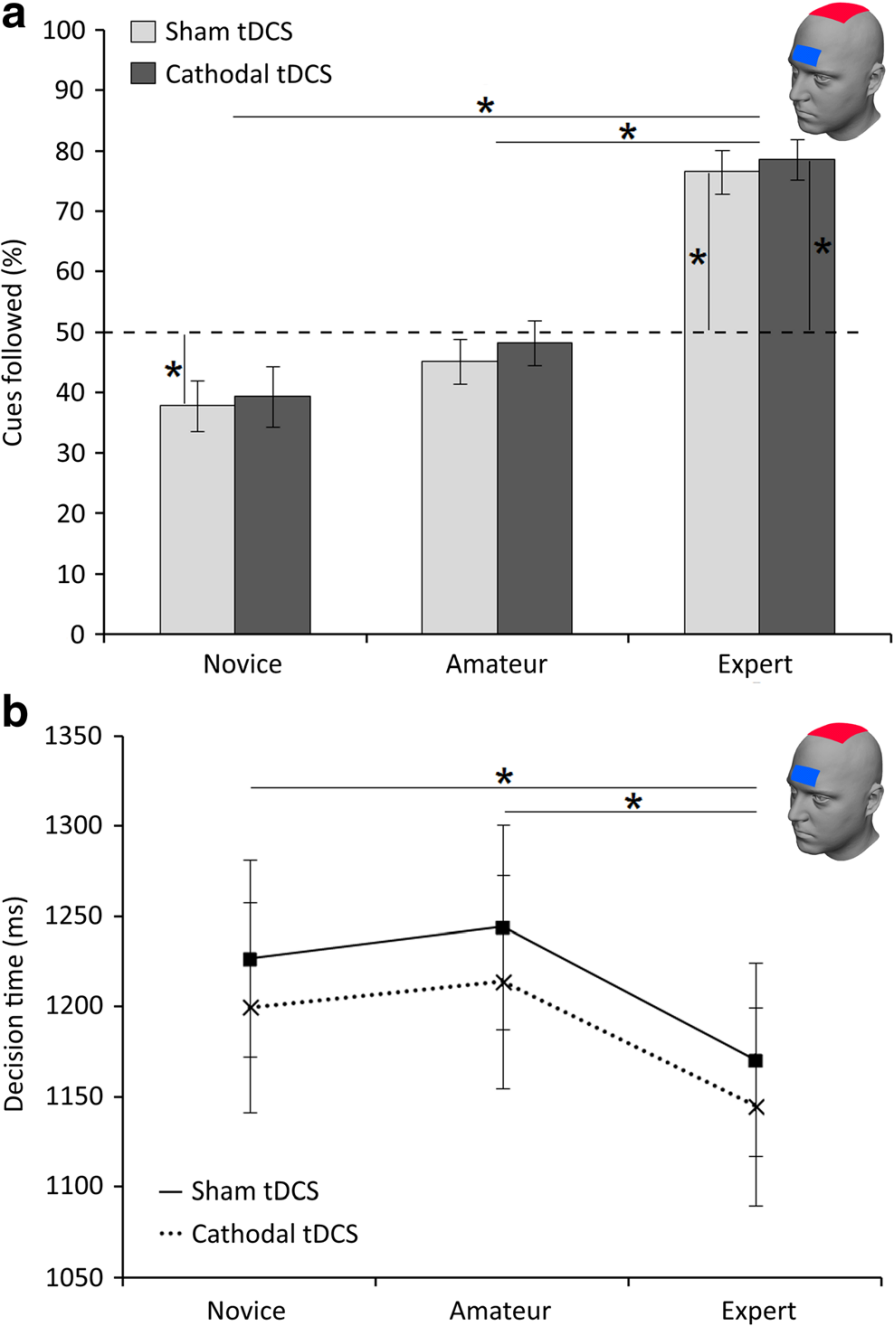
(**a**) Percentages of advice following for novice, amateur, and expert cues during the sham and ctDCS conditions in Experiment 2. Percentages following were compared between the different cue types, between sham and active stimulation, and to chance level (50%). (**b**) Decision times during the sham and ctDCS conditions for each of the three advice cues. Asterisks indicate significant results after Bonferroni correction (*p* < .05)

In the sham condition, participants chose the left vase in 48.44 ± 1.02% of trials and the right vase in 51.36 ± 1.05% of trials, which did not differ significantly [*t*(29) = 1.41, *p* = *.*17], and made responses too late in 0.19 ± 0.08% of trials. In the ctDCS condition, participants chose the left vase in 48.61 ± 0.87% of trials and the right vase in 50.97 ± 0.87% of trials, which also did not differ significantly [*t*(29) = 0.76, *p* = *.*45], and responded too late in 0.42 ± 0.14% of trials. GLM repeated measures ANOVA revealed an effect of advice information on decision making [*F*(2, 58) = 46.24, *p* < .001]. On average, participants followed novices in 38.60 ± 3.50% of trials, amateurs in 46.67 ± 2.76%, and experts in 77.57 ± 2.66%. As in Experiment 1, Bonferroni-corrected pairwise comparisons showed that participants followed the experts significantly more than either the novices [*t*(29) = 7.65, *p* < .001] or the amateurs [*t*(29) = 8.64, *p* < .001], but no significant difference was observed between novices and amateurs (Fig. 4a). Bonferroni-corrected one-sample *t* tests showed that the SPV of novice cues in the sham condition [*t*(29) = – 3.47, *p* = .030], as well as the SPVs of expert cues in both the sham [*t*(29) = 8.60, *p* < .001] and active ctDCS [*t*(29) = 10.23, *p* < .001] conditions were significantly different from chance level. No deviations from chance level were observed after amateur or novice cues during active ctDCS. No main effect of ctDCS condition was found, nor was there an interaction effect between type of cue and stimulation (Fig. 4a). Furthermore, the results cannot be explained by a test–retest effect, because percentage-following behavior did not differ significantly between the first and second sessions [*F*(2, 58) = 0.66, *p* = *.*421].

Decision times also differed significantly between the advice cue categories [*F*(2, 50) = 18.64, *p* < .001]. Bonferroni-corrected pairwise comparisons showed that during expert trials, decision times were significantly faster than during both novice trials [*t*(29) = 4.59, *p* < .001] and amateur trials [*t*(29) = 4.49, *p* = .001], with no difference between novice and amateur trials. Reaction times did not differ between sham and active ctDCS, nor was there an interaction effect between type of cue and stimulation (Fig. 4b). In sum, frontal ctDCS did not influence the use of advice as compared to sham.

#### Effect of ctDCS on reward and punishment ERPs

**Fig. 5:**
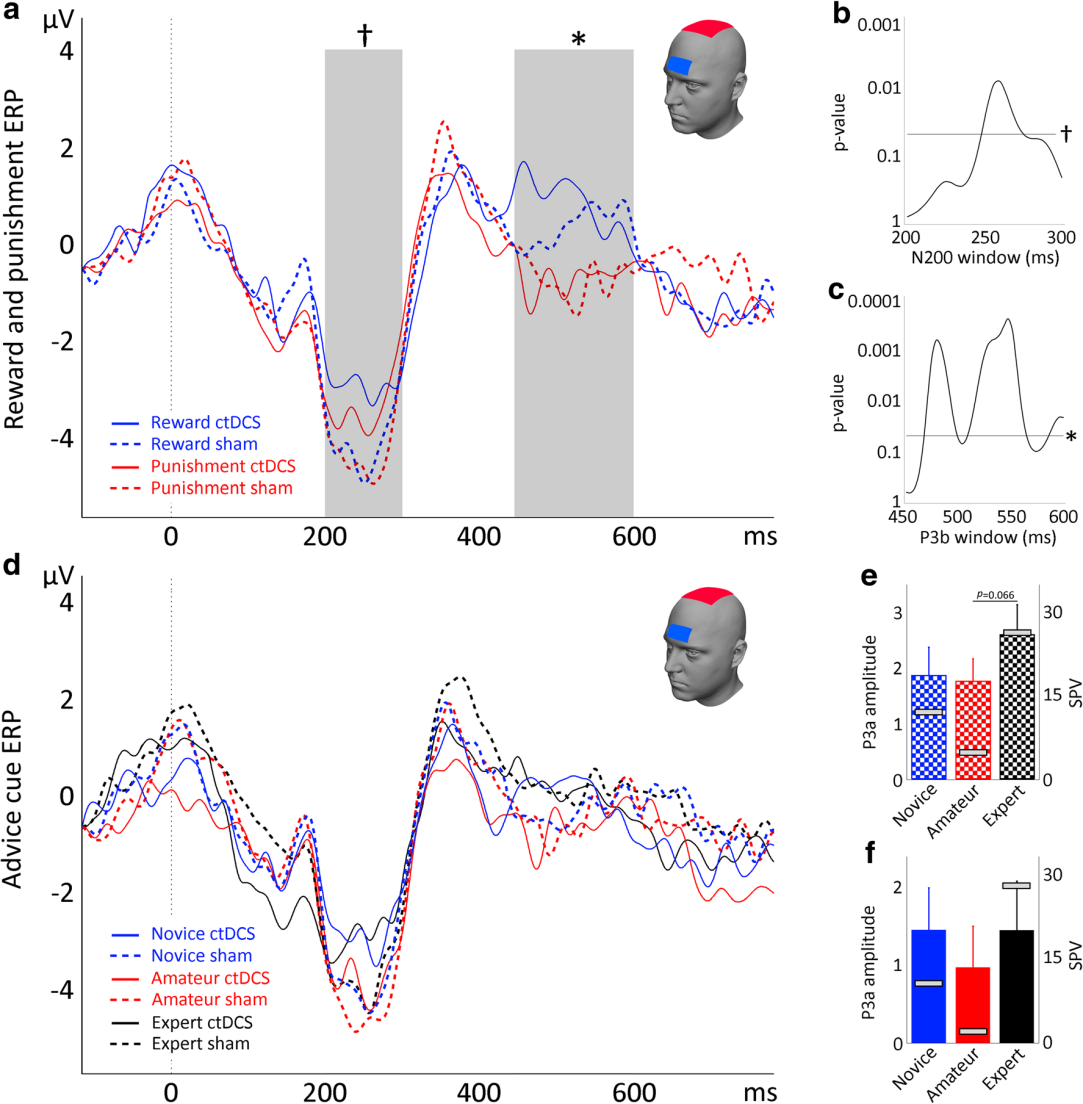
(**a**) ERP responses time-locked to reinforcement feedback immediately after decision making during sham versus ctDCS in Experiment 2. The asterisk indicates a significant effect of RewPun, and the dagger indicates a significant effect of Stim (*p*s < .05). (**b**) Time course (200–300 ms after feedback onset) of the Stim effect. (**c**) Time course (450–600 ms after feedback onset) of the RewPun effect. (**d**) ERP responses time-locked to reinforcement feedback for different advice cues during sham versus ctDCS in Experiment 2. (**e** & **f**) P3a amplitudes (350–400 ms) for advice cues (left *y*-axes), with gray bars reflecting the SPV (right *y*-axes), during (**e**) sham stimulation and (**f**) active ctDCS

As in Experiment 1, analysis of the feedback-locked ERPs revealed a significant effect of RewPun for the P3b [*F*(1, 22) = 12.73, *p* = .001; Fig. 5a]. This effect was specific to a time window between 472 and 568 ms, with reward feedback inducing larger P3b amplitudes than did punishment feedback (Fig. 5c). No significant RewPun effect was observed for the FRN [*F*(1, 22) = 0.87, *p* = .361] or the P3a [*F*(1, 22) = 0.62, *p* = .438].

A significant main effect of Stim (ctDCS vs. sham) was observed for the FRN [on average, *F*(1, 22) = 4.75, *p* = .040; Fig. 5a]. As compared to sham, ctDCS decreased the amplitude of the FRN in the window between 248 and 272 ms (Fig. 5b). No significant Stim effect was observed for the P3a [*F*(1, 22) = 1.55, *p* = .226] or the P3b [*F*(1, 22) = 0.03, *p* = .862]. Also, no significant RewPun × Stim interaction was found for the FRN [*F*(1, 22) = 0.34, *p* = .566], P3a [*F*(1, 22) = 0.52, *p* = .477], or P3b [*F*(1, 22) = 1.07, *p* = .314]. The difference in FRN amplitudes between active and sham ctDCS was not significantly correlated with overall percentages following (*r* = .044, *p* = .842), response times (*r* = – .202, *p* = .354), or overall SPVs (*r* = – .089, *p* = .686).

Furthermore, the mean standard deviations of the ERP signals between 0 and 600 ms were comparable across conditions. In the sham condition, the average standard deviations were 13.19 ± 0.34 *μ*V for the punishment condition and 13.12 ± 0.46 *μ*V for the reward condition. In the active ctDCS condition, the average standard deviation for the punishment condition was 13.81 ± 0.64 *μ*V, and that for the reward condition was 14.05 ± 0.64 *μ*V. A 2×2 within-subjects ANOVA revealed no significant differences between stimulation conditions [*F*(1, 22) = 1.24, *p* = .278], nor between reward and punishment [*F*(1, 22) = 0.19, *p* = .670], nor an interaction effect [*F*(1, 22) = 0.97, *p* = .335]. This suggests that the noise levels did not differ between real and sham ctDCS.

#### Effect of ctDCS on advice cue ERPs

CueType did not affect P3a amplitudes significantly [*F*(2, 44) = 0.45, *p* = .639; Fig. 5d]. However, during sham tDCS the P3a component did show a trend toward modulation by subjective predictive value comparable to that found in Experiment 1 (Fig. 5e), even though the difference between the P3a amplitudes of amateur and expert cues did not reach statistical significance (*p* = .066). Similar though more variable results were observed in the ctDCS condition, indicating that active stimulation did not significantly alter P3a amplitudes (Fig. 5f). As in Experiment 1, no effect of CueType was found on the FRN [*F*(2, 44) = 0.80, *p* = .454] or the P3b [*F*(2, 44) = 0.52, *p* = .596].

As with the reward and punishment trials, a trend toward decreased FRN amplitudes after ctDCS as compared to sham was observed, but this did not reach significance [*F*(1, 22) = 3.12, *p* = .090]. No Stim effect was found for the P3a [*F*(1, 22) = 2.26, *p* = .146] or the P3b [*F*(1, 22) = 0.32, *p* = .579].

No interaction effect of CueType × Stim was observed in the FRN [*F*(2, 44) = 0.42, *p* = .657], P3a [*F*(2, 44) = 0.36, *p* = .703], or P3b [*F*(2, 44) = 0.43, *p* = .655].

#### Effect of ctDCS on evoked power

**Fig. 6:**
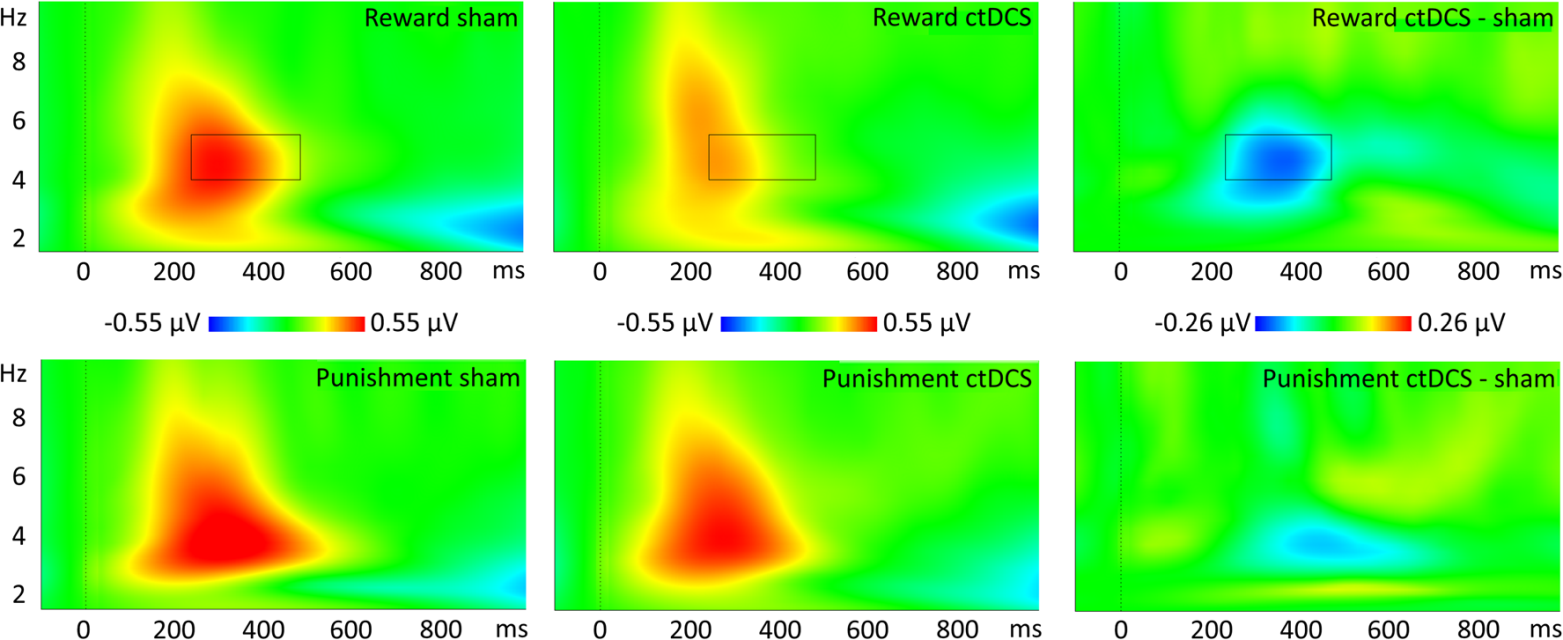
Frequency spectrum plots time-locked to reinforcement feedback immediately after decision making in Experiment 2. In the upper panels, the time–frequency plots are shown for reward trials during sham (left) and active (middle) stimulation, as well as the activity difference (right). In the lower panels, the same plots are shown for punishment trials. The boxes indicate a significant difference between active and sham stimulation (*p* < .05)

An exploratory analysis of event-locked data in the time–frequency domain revealed that real as compared to sham tDCS decreased the activity between 4 and 5.5 Hz in a time window of 200–500 ms after feedback onset (Fig. 6). However, this effect was only observed in reward [*F*(1, 22) = 6.52, *p* = .018] and not in punishment [*F*(1, 22) = 0.78, *p* = .387] trials.

### Discussion

When individuals experience outcome uncertainty during decision making, they tend to rely on advice that may contain relevant information that contributes to a positive outcome. As such, expert advice is considered more credible at face value than advice by nonexperts (Harvey & Fischer, 1997; Van Swol & Sniezek, 2005). In the first experiment we showed that during uncertainty, expert advice is indeed followed significantly more often than amateur and novice advice. Furthermore, participants responded significantly faster to cues that were perceived as being more informative, which is in accordance with previous findings (Klucharev et al., 2011; Meshi et al., 2012).

These observations are in accordance with the prospect theory of decision making (Kahneman & Tversky, 1979). Kahneman and Tversky proposed that feedback signals are used to form an internal inference model (heuristic) to predict which choices will yield expected, better than expected, or worse than expected outcomes. However, uninformative feedback lacks predictive power and introduces uncertainty, and consequently contextual framing effects strongly influence decision making (Tversky & Kahneman, 1992). Due to the low predictability of the reward and punishment feedback, participants start to rely on alternative cues to guide their decisions. In other words, because the reward–punishment feedback cannot be used to form an internal prediction model, participants will become more sensitive to expert advice.

Our first finding was that the FRN was not modulated by feedback cues. The FRN is thought to reflect the discrepancy between predicted and actual outcomes (Holroyd & Coles, 2002). The absence of effects therefore suggests that prediction–outcome mismatch detection is unaffected by advice cues. Importantly, since reward and punishment feedback was presented randomly, participants were not able to form a reliable internal prediction model. As a result, brain signals related to the detection of anticipated versus actual outcomes were not be expected. This is also underlined by the observation that no effect of valence was observed in the FRN. Several studies have suggested that the FRN is affected by valence, with larger amplitudes for negative than for positive feedback (Bellebaum et al., 2010; Wu & Zhou, 2009). Hence, the FRN may distinguish between better- and worse-than-expected outcomes (Alexander & Brown, 2011; Bellebaum et al., 2010; Ullsperger et al., 2014). The absence of a valence effect in this study points toward the absence of a reliable prediction model.

In accordance with prospect theory, during the absence of a reliable internal model, no errors in prediction will be observed, and individuals will rely strongly on external cues instead. Our results showed that, in contrast to the FRN, P3a amplitudes did seem to be affected by advice cues. This may suggest that the subjectively perceived predictability of a cue may be reflected by a change in the P3a amplitude. The least informative amateur’s advice showed the smallest P3a amplitude, whereas the most informative expert cues showed the largest P3a amplitude. Previous studies have shown that the P3a amplitude is associated with the allocation of attentional resources (Polich, 2007). Furthermore, Kopp et al. (2016) showed that P3a amplitudes increase with higher certainty as more attention is allocated toward information that is subjectively perceived as being the most valid. In our study, participants’ certainty depended on the SPV of each advice cue, since the reward and punishment feedback signals were randomly given and thus uninformative. In this context, expert advice is considered to be more reliable than amateur advice, which yields increased attention toward the feedback. Importantly, although the results for the P3a in the second experiment pointed in the same direction, differences between the expert and amateur cues did not reach significance (*p* = .066, two-tailed). The interpretation of the P3a results is therefore tentative and needs to be further explored in future investigations.

In contrast to the P3a component, the P3b was not affected by the SPV of each cue. This is in line with the idea that no internal prediction model was generated, and hence no updating occurred—which is associated with the P3b amplitude—could have occurred. Since the reward and punishment feedback was not predictive, the advice cues were perceived as the primary source of information, and since the cues remained the same over time, no additional learning and, hence, prediction model updating occurred (Chase et al., 2011; Tversky & Kahneman, 1992). This is supported by the observation that participants showed no difference in following advice cues between sessions in the second experiment.

In the second experiment we investigated the contributions of the frontal reward–punishment network that underlie these electrophysiological components by using concurrent ctDCS and EEG. Reinhart and Woodman (2014) showed that offline frontal ctDCS prior to a decision-making task decreases FRN amplitudes and was related to slower learning rates. This suggests that by decreasing the FRN, and hence the detection of prediction errors, the generation of internal predictions models was hindered. Here we showed that the FRN amplitude is also decreased during ctDCS. Furthermore, in our study the ctDCS-induced decrease in the FRN did not influence decision making, further suggesting that the participants tended to rely on the advice and not on reward–punishment feedback. It should also be noted that contrary to Reinhart and Woodman, we investigated the online effects of simultaneous tDCS on the electrophysiological components. Our study therefore provides evidence that tDCS can directly affect the FRN, which is not necessarily related to plasticity effects in the underlying cortical tissue*.* Previous studies have linked the FRN to activity in the theta band (Cavanagh et al., 2014; Hajihosseini & Holroyd, 2013), and it has previously been shown that increased learning speeds are associated with decreased theta activity (Cavanagh, Frank, Klein, & Allen, 2010; Mas-Herrero & Marco-Pallares, 2014; Wischnewski, Zerr, & Schutter, 2016). Indeed, a significant decrease in theta activity in reward trials was observed after ctDCS.

Notably, ctDCS did not modulate the P3a amplitude, which may have been due to the tDCS montage, which may not have targeted the actual neural generator of the P3a component. Another explanation is that ctDCS did target the intended neuronal population for generating the P3a, but that the physiological effects were too small to cause a measurable change in amplitude and behavior. It should be noted, however, that the currently used tDCS montage has been shown to cover a significant portion of the frontal cortex, including the OFC and medial frontal cortex (Manuel et al., 2014). P3a source-localizing studies have identified a broad network within frontal, parietal, and cingulate areas (Sabeti, Katebi, Rastgar, & Azimifar, 2016; Volpe et al., 2007) that broadly corresponds to the so-called *fronto-parietal attention* network (Szczepanski, Pinsk, Douglas, Kastner, & Saalmann, 2013). Although there seems to be considerable overlap between the areas underlying P3a generation and the area being stimulated here with ctDCS, it is unknown whether this montage actually targets the critical subregions and networks. Furthermore, meta-analyses have shown that tDCS effects on cognition are subtle, with a small-to-moderate effect size at best (Dedoncker, Brunoni, Baeken, & Vanderhasselt, 2016; Jacobson, Koslowsky, & Lavidor, 2012). So, whereas ctDCS did influence FRN activity, the P3a was not modulated by the current tDCS montage. As indicated by the observation that the ctDCS-induced change in the FRN was not related to any ctDCS-induced behavioral changes, the FRN does not seem to be part of the subjective valuation of advice cues. However, because the present study was not designed to provide evidence for this null hypothesis, more research will be needed to shed light on whether or not a relationship between the FRN and advice taking exists.

Finally, several limitations should be mentioned. First, the presentation of feedback immediately followed the participant’s button response. This response was given by a button press of the left or right index finger, which may have evoked movement-related potentials in electrodes near the primary motor cortex, specifically C3 and C4. Unfortunately, EEG was not recorded from the C3 and C4 electrodes due to the size of the return electrode. Even though possible contributions of movement-related potentials cannot be completely excluded, due to the equal distributions of left and right responses, it not likely that movement-related potentials would have affected recording from more posterior electrode locations such as the Pz. Second, due to the relatively small number of trials, no interaction between reward/punishment and advice cue on the ERP signals could be analyzed. However, since the effects of advice cues and reward versus punishment were observed in different time windows (350–400 and 450–600 ms, respectively) no interaction effect was expected. Third, the present study suggests that the FRN and P3b have no direct involvement in the processing of advice cues, since decision-making behavior itself was not affected by the ctDCS. The behavioral changes that are associated with FRN modulation using the present ctDCS montage cannot be answered by the present study, because in this study no learning task was used. Finally, combining EEG and tDCS in the present montage was challenging, and due to a low signal-to-noise ratio at the frontal electrodes, we could not analyze the full EEG recordings (Mancini et al., 2015). Channels were excluded if more than 50% of epochs were rejected. Electrode Fz, at which the FRN component is typically measured (Baker & Holroyd, 2011), had to be rejected in 77% of the participants. Therefore, the FRN component was analyzed at the Pz electrode. To the best of our knowledge, this is the first study to have investigated feedback-related ERP components during tDCS, and despite the suboptimal scalp location, a clear negativity was observed between 200 and 300 ms after feedback onset. In addition to a decreased signal-to-noise ratio, a blood pulsation artifact was observed at around 1 Hz (Noury, Hipp, & Siegel, 2016), and therefore a high-pass filter of 1.5 Hz was used, which did not affect the observed difference in the FRNs between real and sham stimulation (Supplementary Fig. S3).

### Conclusion

The present study has shown that during uncertainty, participants direct their attention to expert advice. On the electrophysiological level, this is evidenced by a larger P3a during subsequent reward- and punishment-related feedback processing. Other components, such as the FRN and P3b, were not affected by advice cues. However, ctDCS targeting frontal cortical areas decreased FRN amplitudes. This suggests that advice cues can alter feedback-related electrocortical signals by primarily affecting attentional processes, whereas the detection of and adaptation to mismatches between a prediction model and the actual outcome do not seem to be influenced.

## Compliance with ethical standards

ESM 1 (DOC 3806 kb)
