# Supplementary material for: Frontal cortex electrophysiology in reward- and punishment-related feedback processing during advice-guided decision making: An interleaved EEG-DC stimulation study
Source: Cogn Affect Behav Neurosci. 2018 Jan 29;18(2):249–62. doi: 10.3758/s13415-018-0566-8 (PMC5889418; doi:10.3758/s13415-018-0566-8)
Supplement: ESM 1 — (DOC 3806 kb) [file 13415_2018_566_MOESM1_ESM.doc]

**Supplementary Table S1**

|  | **F1** | **F2** | **Fz** | **F3** | **F4** | **T7** | **T8** | **P3** | **Pz** | **P4** | **O1** | **Oz** | **O2** | **Total loss** |
| --- | --- | --- | --- | --- | --- | --- | --- | --- | --- | --- | --- | --- | --- | --- |
| **Pp 1** | A+S | A+S | A |  | A |  |  | A |  |  |  |  |  | 5 |
| **Pp 2** | A | A | A | A | A | A | A | A | A | A | A | A | A | 13 |
| **Pp 3** |  |  |  |  |  |  |  |  |  |  |  |  |  | 0 |
| **Pp 4** | A+S | A+S | A+S |  | A |  |  |  |  | A |  |  |  | 5 |
| **Pp 5** | S | S |  |  | S |  |  |  |  |  |  |  |  | 3 |
| **Pp 6** | S | A+S | S | A | A |  |  |  |  | A |  |  |  | 6 |
| **Pp 7** | A |  | A | A | A |  |  | A |  | A |  |  |  | 6 |
| **Pp 8** | S | S |  |  |  |  |  |  |  |  |  |  |  | 2 |
| **Pp 9** | A | A | A | A | A |  |  | A | A | A |  |  |  | 8 |
| **Pp 10** | A+S | A+S | A+S | A+S | A |  |  |  |  |  |  |  |  | 5 |
| **Pp 11** |  | A | A |  | A |  |  |  |  |  |  |  |  | 3 |
| **Pp 12** | A | A | A |  |  |  |  |  |  |  |  |  |  | 3 |
| **Pp 13** | A | A+S | A | A | A |  |  | A | A | A |  |  |  | 8 |
| **Pp 14** | A+S | A+S | A | A+S | A |  |  |  |  |  |  |  |  | 5 |
| **Pp 15** |  |  |  |  |  |  |  |  |  |  |  |  |  | 0 |
| **Pp 16** | A | A | A | A | A |  |  |  |  |  |  |  |  | 5 |
| **Pp 17** | S | A | A | A |  |  |  |  |  |  |  |  |  | 4 |
| **Pp 18** | S | S | S | S |  |  |  |  |  |  |  |  |  | 4 |
| **Pp 19** |  |  |  |  |  |  |  |  |  |  |  |  |  | 0 |
| **Pp 20** |  | S | A |  |  |  |  |  |  |  |  |  |  | 2 |
| **Pp 21** | S | A+S | S | S | S | S | S | S | S | S | S | S | S | 13 |
| **Pp 22** | A | A+S | A | A | A | A | A | A | A | A | A | A | A | 13 |
| **Pp 23** |  | A+S | A+S | A+S |  |  |  |  |  |  |  |  |  | 3 |
| **Pp 24** | A+S | A | A | A | A |  |  |  |  |  |  |  |  | 5 |
| **Pp 25** | A+S | A | A+S | A+S |  |  |  |  |  | A+S |  |  |  | 5 |
| **Pp 26** |  | A+S | A | A | S |  |  |  | A | A |  |  |  | 6 |
| **Pp 27** | A | S | A | A | S |  |  |  | A+S |  |  |  |  | 6 |
| **Pp 28** |  |  |  |  |  |  |  |  |  |  |  |  |  | 0 |
| **Pp 29** |  | S | S | S | S |  |  |  |  |  |  |  |  | 4 |
| **Pp 30** |  |  |  |  |  |  |  |  |  |  |  |  |  | 0 |
| **Total loss** | 20 | 24 | 23 | 18 | 18 | 3 | 3 | 7 | 7 | 10 | 3 | 3 | 3 |  |

EEG channels that were too noisy due to artifacts produced by the tDCS apparatus are indicated by the letter A or S. Data loss was observed during active stimulation (A) and during sham stimulation (S). The recording was discarded > 50% of the data was rejected due to artifacts. Data loss was mainly observed in channels that were close to the tDCS electrodes in the frontal channels. Furthermore, artifacts were not only limited to active stimulation, but were also observed during sham stimulation. Although during sham stimulation no active current is flowing, the DC-stimulator is turned on. A decrease in signal-to-noise ratio of EEG signals was observed in a number of participants due to small passive current leakage from the stimulator.

**Supplementary Figure S1**

**
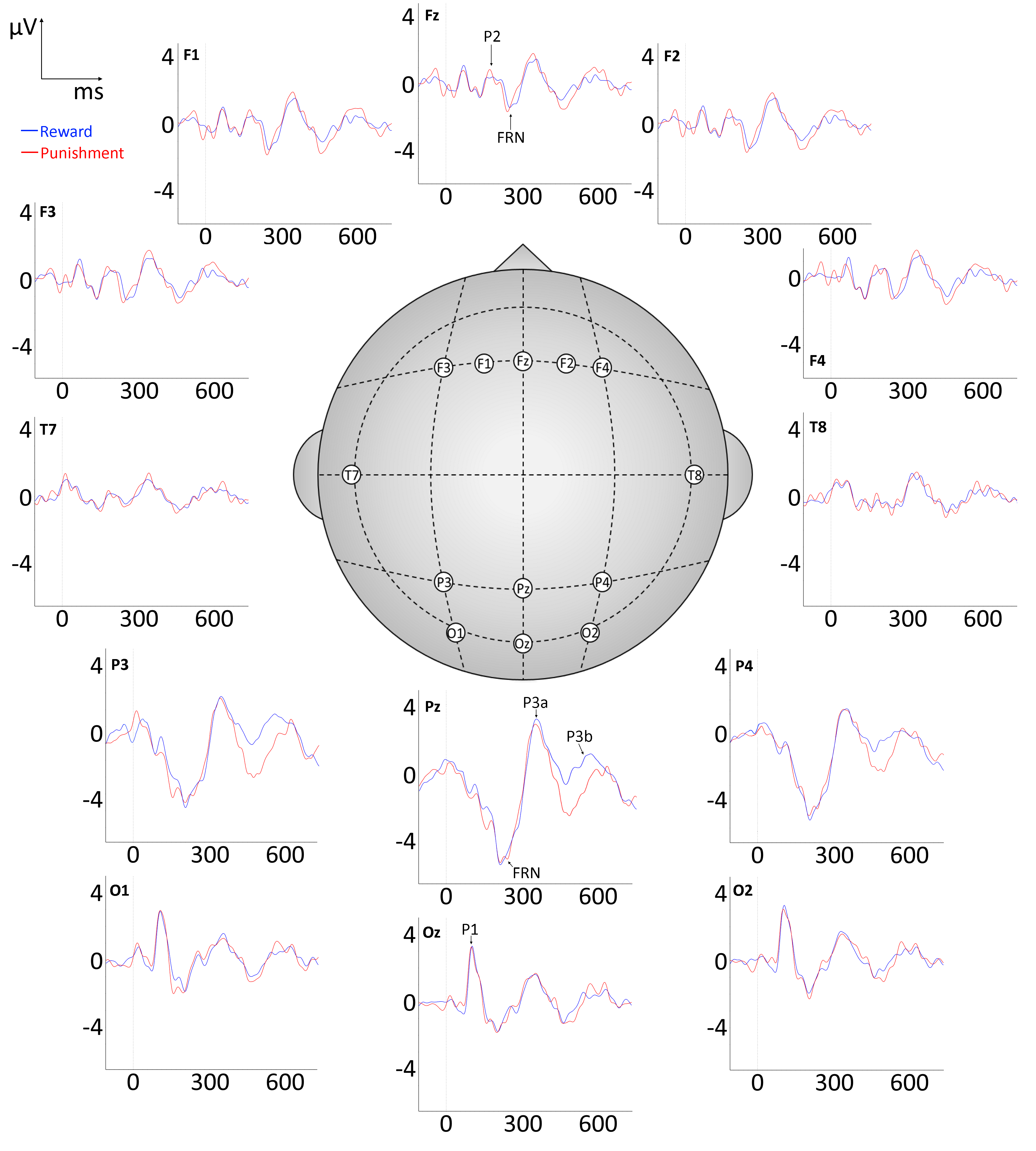
**

An overview of ERPs after feedback onset (0 ms) in all recorded channels for reward and punishment trials. Around 100 ms a positive peak (P1) is observed in occipital electrodes. A second positive peak (P2) is observed at the frontal electrodes around 200 ms, followed by a fronto-parietal negativity (FRN). Lastly a positive peak is observed around 375 ms (P3a), which is followed by a relative positivity, referred to as P3b (450-600 ms). A significant main effect of observed for the P3b component (450-600 ms) in electrode Pz (*F(1,20)* = 5.99, *p* = .024). The same effect was also observed in electrode P3(*F(1,20)* = 10.41, *p* = .004) and P4 (*F(1,20)* = 7.05, *p* = .015). None of the other channels showed a significant difference in FRN, P3a or P3b.

**3.5-30 Hz**

**Supplementary Figure S2**

**
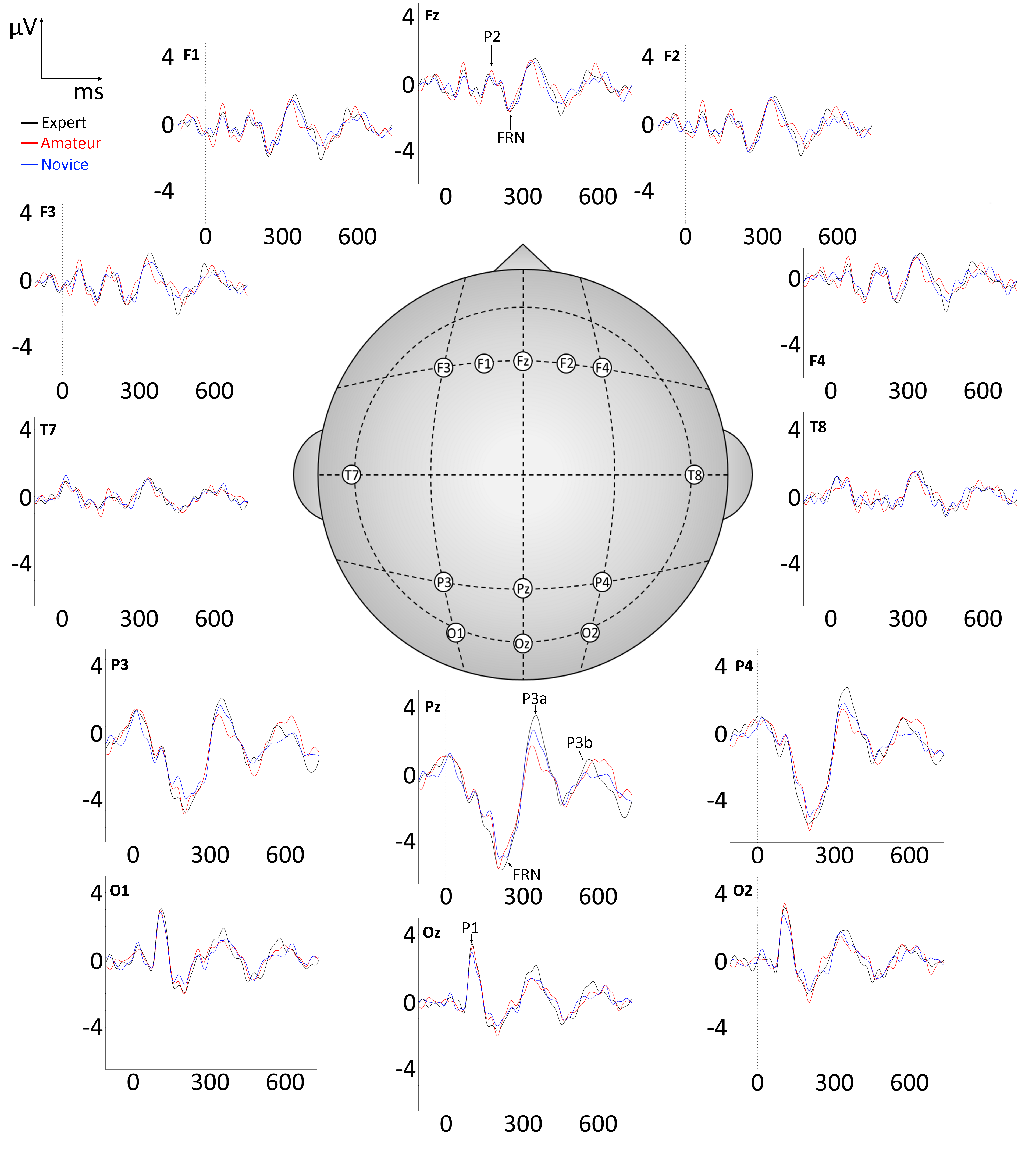
**

An overview of ERPs after feedback onset (0 ms) in all recorded channels for novice, amateur and expert cues. A significant main effect of observed for the P3a component (350-400 ms) in electrode Pz (*F(2,40)* = 3.30, *p* = 0.047). None of the other channels showed a significant difference in FRN, P3a or P3b.

**3.5-30 Hz**

**Supplementary Figure S3**

**
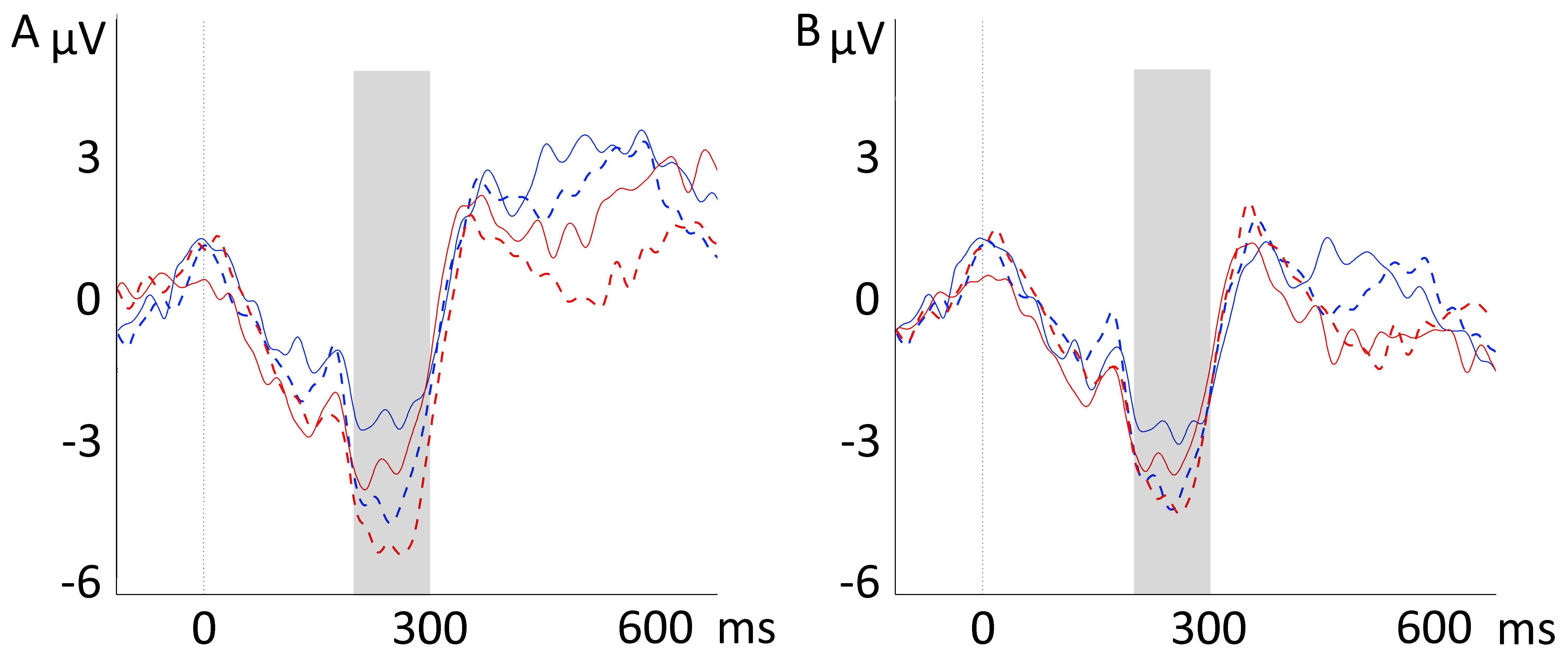
**

For the offline analysis of ERP components a band-pass filter of 1.5-30 Hz was used. Frequencies below 1.5 Hz were discarded due to the possibility of an artifact caused by blood pulsation in skin arteries (Noury et al., 2016). However, as feedback-related ERPs are driven by low-frequency oscillations in the delta and theta range (Cavanagh et al., 2014), it is possible that this filter setting distorted our results. Therefore, analysis was performed using a band-pass filter of 0.1-30 Hz (Figure S3a). As can be observed, the general waveform differs between this analysis and the original analysis (1.5-30 Hz; Figure S3b). We explored whether the differences in ctDCS vs sham that were observed for the FRN (200-300 ms) could be explained by different filter settings. A GLM repeated measures ANOVA with FRN amplitude (200-300 ms) as dependent variable, and RewPun (reward vs punishment), Stim (ctDCS vs sham) and Filter (0.1-30 Hz vs 1.5-30 Hz) as independent variables was performed. The results showed neither a main effect of Filter (*F*(1,22) = 3.14, *p* = 0.091), nor a Filter*Stim (*F*(1,22) < 0.01, *p* = 0.957), and Filter*Stim*RewPun interaction (*F*(1,22) = 0.30, *p* = 0.590),. This means that the observed difference at the FRN was not distorted due to the filter settings

**References**

Cavanagh, J.F., Masters, S.E., Bath, K., Frank, M.J. (2014). Conflict acts as an implicit cost in reinforcement learning. *Nature Communications*, *5*, 5394.

Noury, N., Hipp, J.F., Siegel, M. (2016). Physiological processes non-linearly affect electrophysiological recordings during transcranial electrical stimulation. *NeuroImage, 140,* 99-109.
